# Simulation of Dispersion and Explosion Characteristics of LiFePO_4_ Lithium-Ion Battery Thermal Runaway Gases

**DOI:** 10.1021/acsomega.3c08709

**Published:** 2024-04-04

**Authors:** Mingjie Zhang, Kai Yang, Qianjun Zhang, Hao Chen, Maosong Fan, Mengmeng Geng, Bin Wei, Bin Xie

**Affiliations:** † China Electric Power Research Institute, Beijing 100192, China; ‡ Jescom Software (Shanghai) Co., Shanghai 200090, China

## Abstract

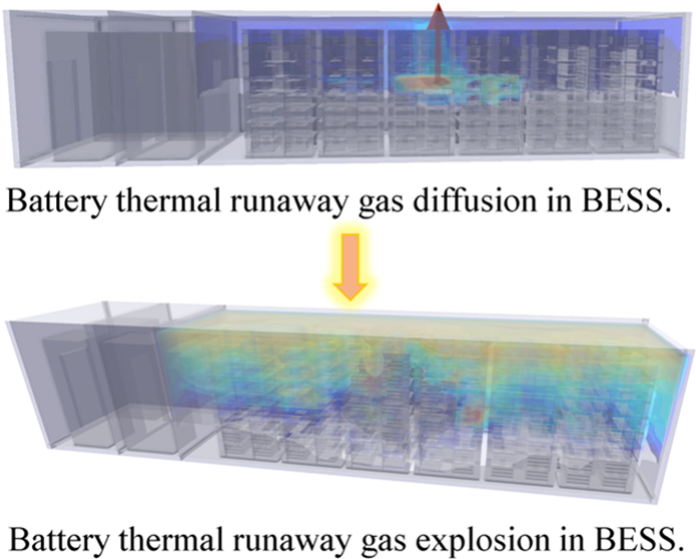

In recent years, as the installed scale of battery energy storage systems (BESS) continues to expand, energy storage system safety incidents have been a fast-growing trend, sparking widespread concern from all walks of life. During the thermal runaway (TR) process of lithium-ion batteries, a large amount of combustible gas is released. In this paper, the 105 Ah lithium iron phosphate battery TR test was conducted, and the flammable gas components released from the battery TR were detected. The simulation tests of the diffusion and explosion characteristics of lithium iron phosphate battery’s (LFP) TR gases with different numbers and positions in the BESS were carried out using FLACS simulation software. It was found that the more batteries TR simultaneously, the shorter the time for the combustible gas concentration in the energy storage cabin to reach the explosion limit. When 48 batteries were in TR simultaneously in the energy storage cabin, the shortest time was 9.8 s, and the further the location of the fire is from the hatch, the largest explosion overpressure is generated to the hatch, up to 583 kPa. When the gas generated by the TR of 48 batteries explodes, the maximum explosion overpressure at 5 m outside the energy storage cabin hatch is more significant than 40 kPa, which will cause serious injury to humans. The causes of TR of batteries in prefabricated chambers are complex, and the location and amount of thermal runaway of batteries as well as the diffusion of combustible fumes can have different effects on the external environment. The research results can provide support for the safety design of BESS.

## 1 Introduction

In the contemporary era marked by the swift advancement of green energy, the progression of energy storage technology attracts escalating attention.^1−3^ Lithium-ion batteries have emerged as a novel electrochemical energy storage approach within this domain, renowned for their extended lifespan and superior energy density. These attributes have facilitated their extensive application in global energy storage initiatives. Recent years have witnessed a shift in lithium-ion battery research from individual units to GWh-scale battery energy storage systems (BESS).^4,5^ Despite these advancements, lithium-ion batteries, under specific internal and external stimuli, are susceptible to thermal runaway (TR) reactions,^6,7^ leading to the substantial release of flammable gases and heightening the risk of fire or explosive incidents.^8−10^ The Beijing 4·16 Dahongmen Energy Station fire accident notably underscored these hazards, propelling industrywide scrutiny of the combustion and explosion characteristics of lithium-ion BESS.

In the realm of gas production from lithium battery TR, extensive research has been conducted.^11,12^ Numerous studies have identified the primary gases produced during battery TR as H_2_, CO, CO_2_, CH_4_, C_2_H_6_, C_2_H_4_, C_3_H_8_, among others.^13,14^ Research by Koch and others^15^ highlights the battery’s capacity and energy density as pivotal factors, influencing gas release, TR initiation temperature, and mass loss. A fluid dynamics model developed using OpenFOAM by Kong and colleagues^16^ reveals the impact of the battery’s State of Charge (SOC) on the onset time and peak jet speed of gas during TR.

In the aspect of lithium-ion battery combustion and explosion simulations, Zhao ’s work^17^ utilizing FLACS software provides insight into post-TR battery behavior within energy storage cabins. The research underscores the significant influence of the ignition point location, environmental temperature, and cabin filling degree on explosion characteristics. Additional research by Jin and others,^18^ equating vaporized electrolytes to combustible gas, emphasizes the critical need for timely management of flammable gases released during battery TR. The study indicates that a single battery module’s gas release can instigate an explosion in energy storage cabins, with concurrent impact on adjacent cabins. Investigations by Xu and others^19^ into the diffusion of TR gases within prefabricated cabins reveal consistent gas component levels at identical cabin heights. Explosion simulation experiments by Yin and others^20^ demonstrate a notable increase in explosion temperature and maximum overpressure with H_2_ involvement.^21,22^

In summation, while extensive research has been conducted on the diffusion and explosion laws of battery TR gases within BESS, a significant research gap exists in the exploration of the combustion and explosion characteristics of gases released during lithium-ion battery TR. This study endeavors to bridge this gap by conducting a comprehensive simulation study on the combustion and explosion characteristics of TR gases from lithium iron phosphate batteries within BESS. Utilizing the mixed gas components generated by a 105 Ah lithium iron phosphate battery (LFP) TR as experimental parameters, and employing FLACS simulation software,^23^ a robust diffusion–explosion simulation model is established. This research meticulously examines the influence of TR quantity and location, offering a comprehensive analysis and summary of the diffusion and explosion patterns of TR flammable gases within BESS, alongside a detailed hazard degree analysis.

## 2 Experimental Method

This study employs a 105 Ah LFP, manufactured by a specific company, as the experimental sample. The parameters of LFP in this experiment are shown in Table 1. The battery samples were charged to full capacity using the constant current (CC) charging method at a rate of 0.3C. The battery operates at a State of Health (SOH) and State of Charge (SOC) of 100% and possesses an energy capacity of 355 Wh. Its aluminum casing is equipped with a safety valve designed to burst at a pressure of 0.8 MPa. The experiment utilizes a fixed-volume pressure vessel, comprising an explosion chamber, pressure gauge, thermometer, exhaust pipeline, and internal circuitry, all engineered to withstand an explosion overpressure up to 5 MPa. This setup enables the real-time collection of various parameters throughout the entire battery TR process.

**Table 1:** Basic Parameters of the Experimental Battery

parameters	value/composition
geometric dimensions L × W × H (mm × mm × mm)	130 × 36 × 195
mass/kg	2.0
rated voltage/V	3.2
rated capacity/Ah	105
electrolyte	LiPF_6_, ethylene carbonate (EC), methylene carbonate (EMC), propylene carbonate (PC), vinylidene carbonate (VC)
anode	graphite
cathode	LFP

**Figure 1:**
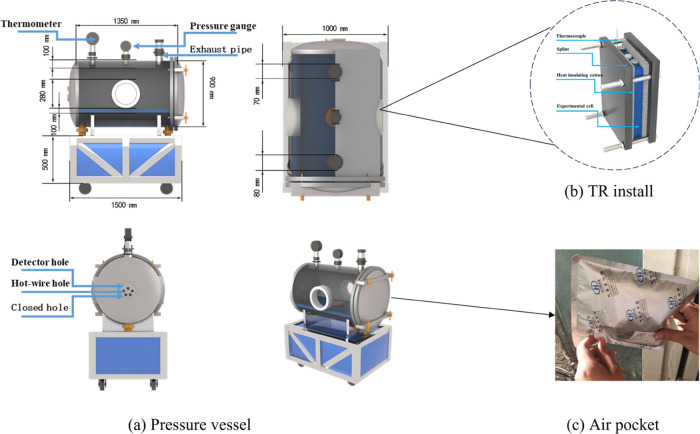
105 Ah LFP TR combustible gas collection and test.

For the heating process, two 500 W heating plates are employed to heat the 105 Ah LFP. K-type thermocouples continuously monitor the battery’s surface temperature, as depicted in Figure 1(b). Prior to the experiment, the pressure vessel is evacuated and filled with argon (Ar_2_), a process repeated thrice. The experiment is halted upon observing a sudden voltage drop between the battery electrodes. Postexperiment, the data recorder is used to extract pertinent voltage and temperature data. A gas collection bag captures the TR gases for analysis using a gas chromatograph (German Exactive GC) to ascertain gas composition and ratios, as depicted in Figure 1(c).

**Figure 2:**
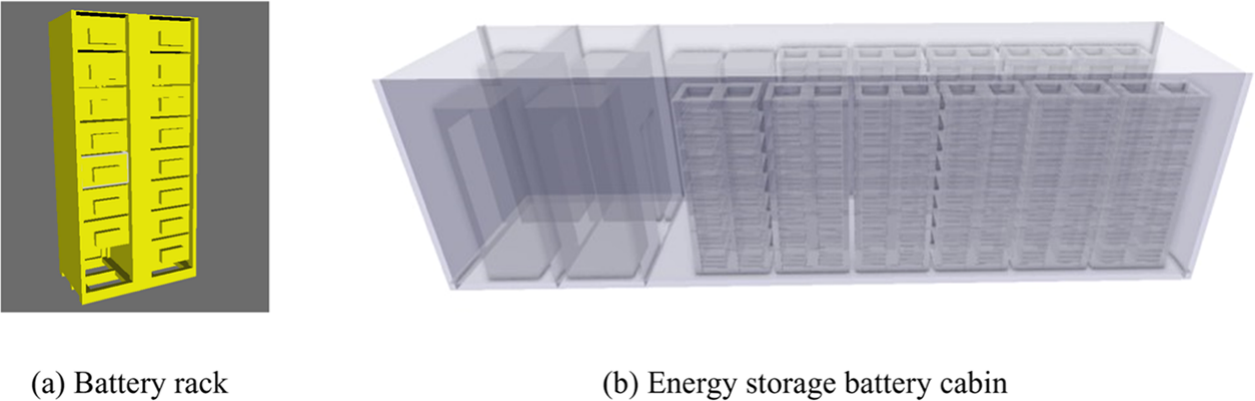
Schematic of the internal structure of the energy storage cabin.

The study utilizes a 40 ft energy storage prefabricated cabin from a specific company as the research object. The prefabricated cabin model, divided into a battery cabin and a control room, houses batteries, each with a capacity of 105 Ah. Each module within the battery cabin contains 48 individual batteries, arranged in 1 parallel and 48 series configurations. The cabin contains a total of 11 battery clusters, with a total system energy of 1.42 MWh and a volume of 31.093 m^3^ with a void ratio of 54.86%. A diffusion–explosion simulation model is established within FLACS simulation software, as illustrated in Figure 2. The input parameters to the model are the TR gas composition, gas release rate, and experimental temperature and pressure, and the materials used in the model are all nondeformable and rigid materials. In the grid design, the spacing between the grids in the interior space of the BESS module is 0.1 m, and the spacing between the grids in the exterior space of the container is enlarged by a factor of 1.25, with a total of 296,000 grids in the BESS module. FLACS simulation model assumptions are as follows:

1. The material in the battery compartment is an indestructible rigid body.
2. The combustible gases released when a battery undergoes TR are well-mixed gases.
3. The cells are closely spaced on the battery rack with no gaps, thus simplifying the battery module to a rectangular shape.
4. There was no exchange of the material between the start of TR in the BESS module and the explosion that destroyed the hatch.
5. The gas environment outside the BESS module is steady-state air at room temperature and pressure.
6. This model does not deal with the heat-spreading process between the cells and simplifies the situation.

FLACS simulation software for numerical calculations in the diffusion and explosion scenarios uses the boundary conditions of the Eulerian boundary, the pressure at the boundary is the ambient pressure, and the continuity equation at the boundary is calculated at atmospheric pressure.

**Figure 3:**
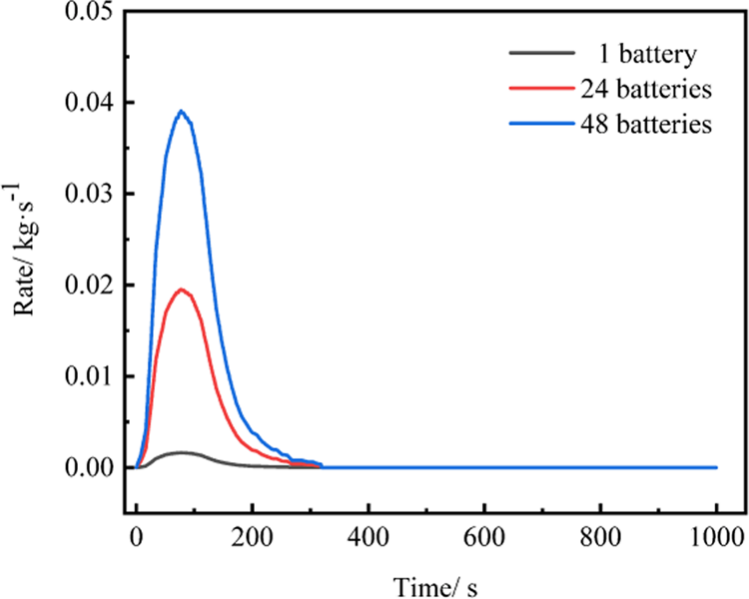
Gas release rate of LFP.

Simulation work is conducted in the energy storage prefabricated cabin, adhering to the gas release rules observed during the TR experiment of LFP.^24^ The gas release rules for 24 and 48 lithium iron phosphate batteries undergoing TR were calculated, as shown in Figure 3, with the gas release process lasting for 310 s.

**Figure 4:**
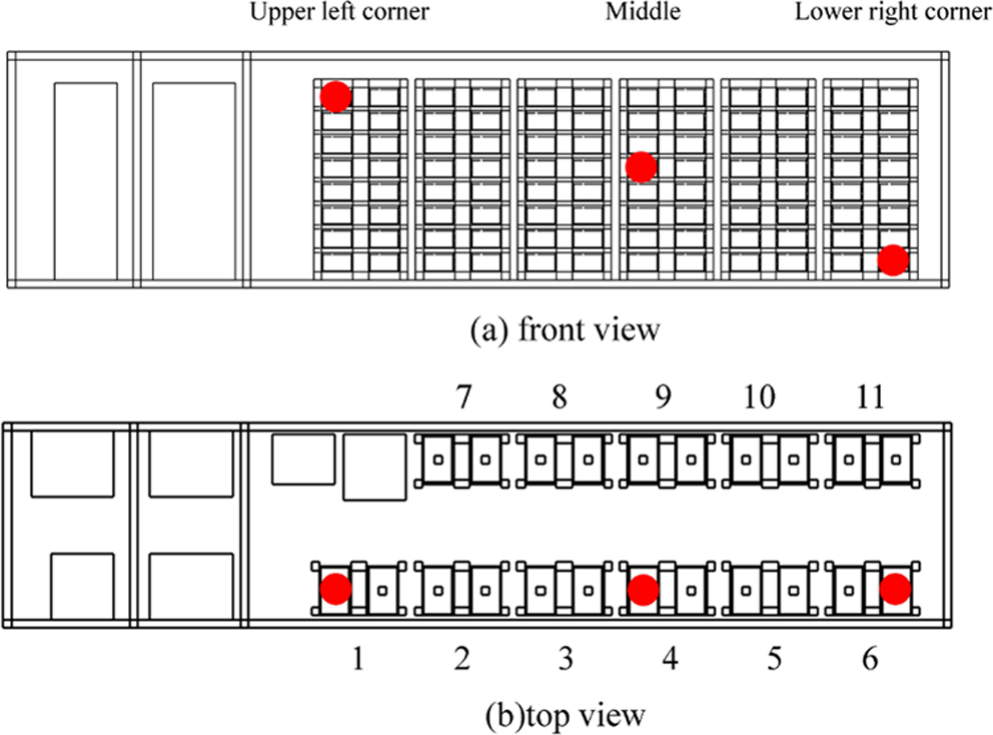
Locations of TR batteries: (a) front view and (b) top view.

The study examines different quantities and positions of battery TR gas diffusion–explosion rules within the prefabricated cabin. The content of the 105 Ah LFP TR gas simulation is outlined in Table 2. The battery TR locations within the prefabricated cabin are identified as the upper left, middle, and lower right positions of the battery cluster, as depicted in Figure 4, where numbers 1–11 represent each battery cluster.

**Table 2:** Content of TR Gas Simulation of LFP

test 1 different quantity		test 2 different position	
number of batteries	TR position	number of batteries	TR position
1 battery	middle	48 batteries	upper left corner
24 batteries	middle	48 batteries	middle
48 batteries	middle	48 batteries	lower right corner

**Figure 5:**
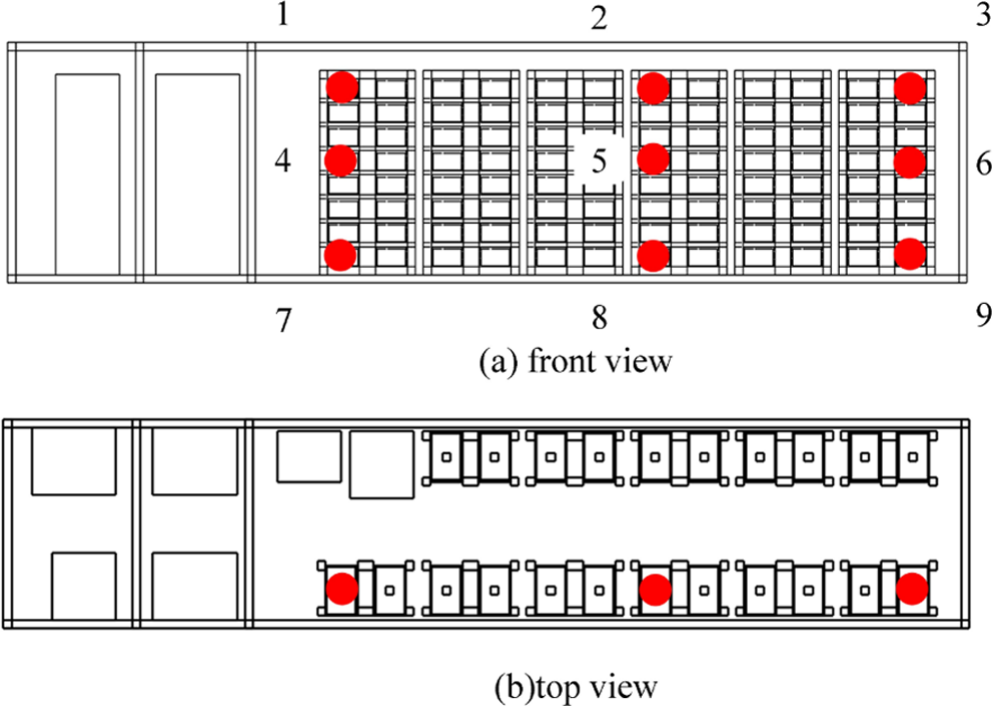
Distribution of ignition positions.

At 1000 s of anions into the battery TR, the mixed flammable gas achieves a stable distribution within the prefabricated cabin. To investigate the explosion rules at various ignition positions and times during the battery TR process, ignition experiments are conducted every 100 s during the 1000 s process of battery TR gas diffusion. Nine positions are selected on the section in the middle position of the battery cluster on one side of the prefabricated cabin, and ignition experiments are conducted at each position at each time point, as shown in Figure 5. The explosion hazard is characterized by the explosion overpressure received by the cabin door on the right side of the prefabricated cabin, which is replaced by a pressure relief plate set to fail when the overpressure on both sides exceeds 15 kPa.

FLACS software is utilized for numerical research on diffusion–explosion within the energy storage prefabricated cabin, solving the Navier–Stokes (N–S) equation on a three-dimensional structural grid using the ideal gas state equation and the K–ε finite element volume method. All parameters during the explosion process adhere to the relevant laws of mass conservation, momentum conservation, and energy conservation. The form of the control equation is generally as follows
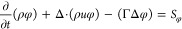
1Due to the intense chemical reactions during the explosion process, the mass fraction during the explosion satisfies the following equation
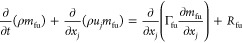
2

## 3 Results and Discussion

### 3.1 Outcomes of Gas Generation Experiment

**Figure 6:**
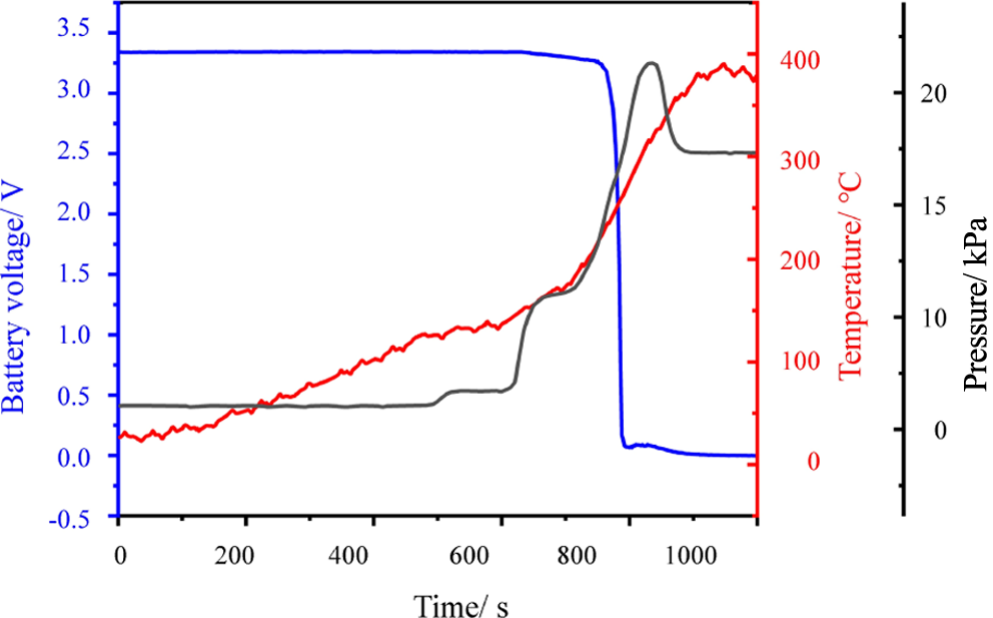
Battery TR pressure, temperature, and voltage variation plots.

**Figure 7:**
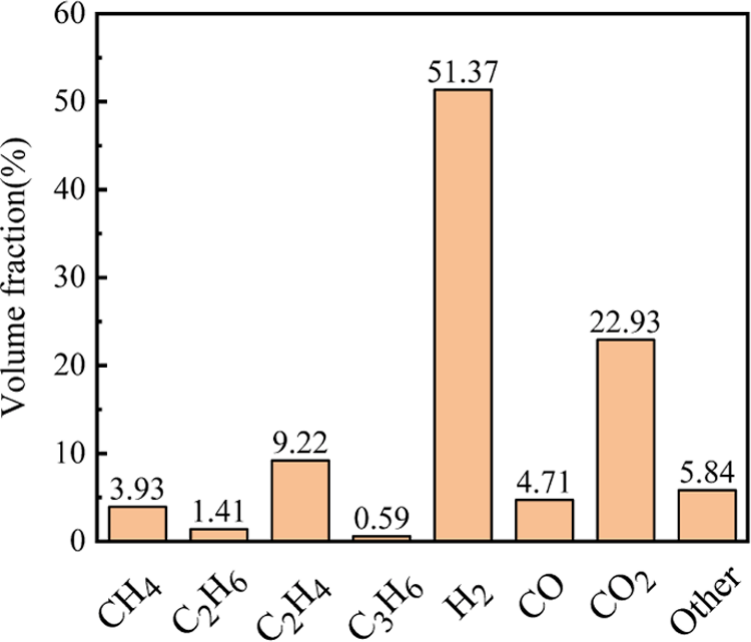
Battery TR gas volume fraction.

During the TR of the battery, alterations in the gas temperature, surface temperature of the battery, and voltage within the pressure vessel are depicted in Figure 6. The apex pressure within the pressure vessel reaches 22 kPa. Utilizing the volume of the pressure vessel and the ideal gas equation, it is inferred that the TR of the 105 Ah LFP generates a gas volume of approximately 85.3 L, weighing around 83.5 g, equivalent to 3.81 mol at standard temperature and pressure. Gas chromatography measurements, as illustrated in Figure 7, indicate that H_2_ comprises over 50% of the combustible gases emitted during the 105 Ah LFP TR. The explosion limit range of this mixed gas, as calculated through the refined Le Chatelier formula,^25^ spans from 4.86 to 52.2%.

This study investigates the TR and venting behavior of 105 Ah commercial Li-ion batteries under overcharging and overheating conditions. The current work and previous studies are summarized in Table 3. Some key parameters of TR are listed in detail. The gas composition of the thermal runaway of Li-ion batteries under overcharging and overheating conditions is analyzed. It reveals the gas generation mechanism of LFP batteries in battery storage systems under overcharging and overheating conditions. The current work is basically similar to previous studies of gas composition. It provides reference data for the fire protection design and accident emergency response of battery energy storage systems.

**Table 3:** Summary of the Research on the TR of LFP Batteries and This Work

reference	battery capacity	abuse test	atmosphere	gas detected
Yang^29^	1.5 Ah	thermal abuse	air	CO_2_, H_2_, CO, CH_4_, C_2_H_4_, C_2_H_6_, C_3_H_6_, C_3_H_8_
Fernandes^30^	2.5 Ah	overcharge	air	H_2_, CO, CO_2_, CH_4_, C_2_H_4_, C_2_H_6_, C_3_H_6_ C_2_H_6_O, C_2_H_5_F, C_2_H_4_O_2_, C_2_H_6_O, CH_4_O, HF
Yuan^31^	3.8 Ah	thermal abuse	air	H_2_, CO, CO_2_, CH_4_, C_2_H_2_, C_2_H_4_, C_2_H_6_
Sturk^32^	7 Ah	thermal abuse	nitrogen	CO_2_, CO, organic carbonates, fluorinated, hydrocarbons, HF, POF_3_
Liu^33^	22 Ah	thermal abuse	air	H_2_, CO_2_, CO, HF
Jia^34^	86 Ah	overcharge/thermal abuse	air	H_2_, CO_2_, CO, C_2_H_4_, CH_4_
Shen^35^	304 Ah	thermal abuse	nitrogen	H_2_, CO, CO_2_, C_2_H_4_, CH_4_
this work	105 Ah	thermal abuse	nitrogen	H_2_, CO_2_, C_2_H_4_, CO, CH_4_, C_3_H_6_, C_2_H_6_, C_2_H_2_

The mass of gas produced by TR of LFP at different SOH^26^ and SOC^27,28^ is different. It was found that as SOC and SOH increase, the battery contains more energy; more flammable gases will be released when the battery undergoes TR during the heating process, and the battery TR time will be extended. In the simulation, if the gas-producing mass and gas-producing time of the batteries are prolonged, there will be a potential for a larger-scale ignition and explosion hazard within the BESS. In this study, the mass and composition of the TR gas of 105 Ah LFP with 100% SOC were used for simulation.

### 3.2 Simulation of Varied Battery Quantities in TR

**Figure 8:**
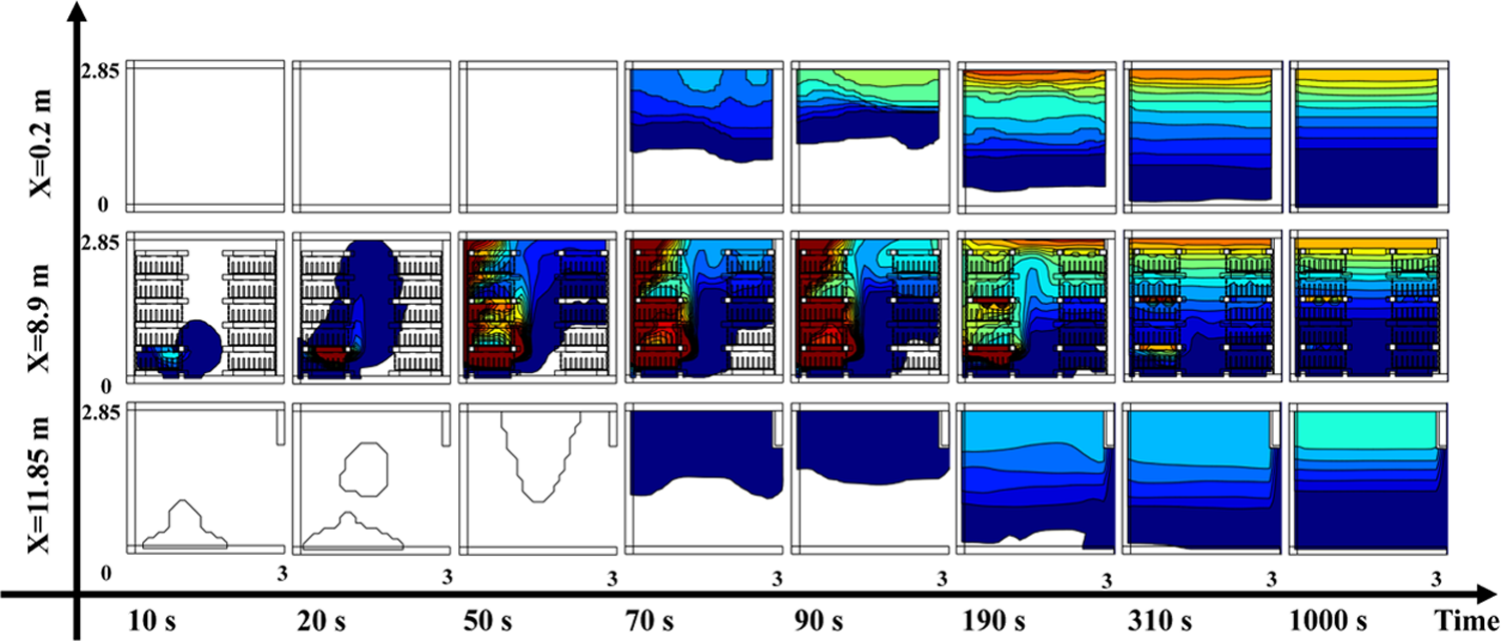
Battery TR gas volume fraction.

To delineate the gas diffusion process within the BESS, this study selects sections at *x* = 0.2 8.9, and 11.85 m within the cabin for examination of gas diffusion during battery TR. These positions correspond respectively to the edge wall of the battery cabin, the cross section at the TR location, and the edge wall of the control room. Figure 8 visually presents the gas diffusion within the prefabricated cabin. To enhance the accuracy of representing gas diffusion variations during battery TR, the chromatogram employs diverse colors to signify different gas concentration ranges, with deeper blues and reds indicating concentrations nearing 0% and higher concentrations, respectively.

As observed in Figure 8, a mere 10 s into the gas diffusion process of battery TR, the mixed gas has pervaded to the outermost edge of the control room. By 20 s, the mixed gas has ascended to the zenith of the prefabricated cabin, forming a gas cloud within the explosion limit range at 50 s. At 190 s, the entire cabin space is inundated with gas emanated from the battery TR. By 310 s, aside from a minor accumulation of gas within the battery module, much of the gas has coalesced into a concentration gradient gas cloud at the upper echelon of the prefabricated cabin. At the 1000 s mark, the gas cloud concentration distribution within the cabin attains stability, with the entire space manifesting a stratified combustible gas cloud.

**Figure 9:**
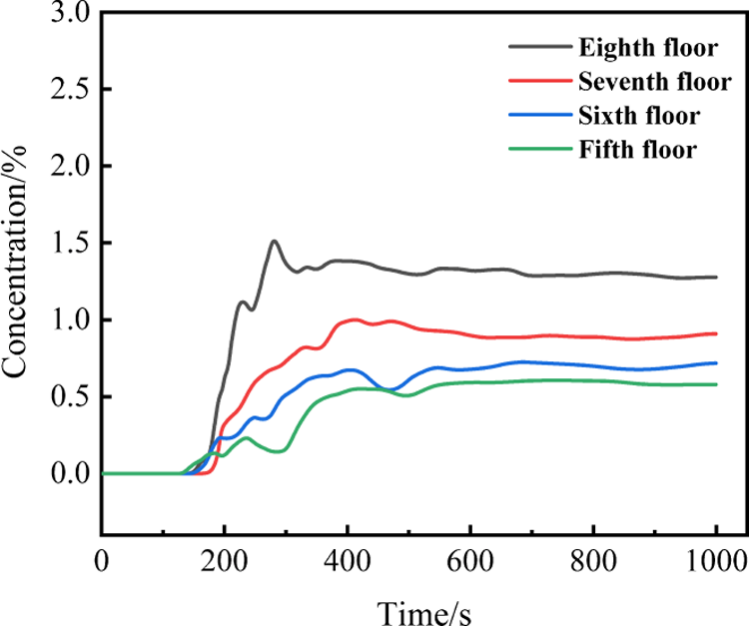
Gas concentrations of different battery rack level.

A battery in TR discharges a total gas mass of approximately 83.5 g, equivalent to a volume of 85.3 L. Figure 9 depicts the fluctuation in combustible gas concentration within the battery cluster experiencing TR, with each layer of the battery module in the upper part of the battery cluster registering a combustible gas concentration below the 4.86% explosion lower limit, thereby negating the possibility of an explosion.

For 24 and 48 batteries concurrently undergoing TR, the total gas mass released is 2 and 4 kg, respectively, correlating to volumes of 2047 and 4094 L. Due to the lower density of the emitted gas compared to air, post-TR, the combustible gas accumulates at the upper segment of the prefabricated cabin. When the combustible gas concentration at a specific location within the cabin surpasses 4.86%, it enters the explosion limit, rendering it ignitable.

**Figure 10:**
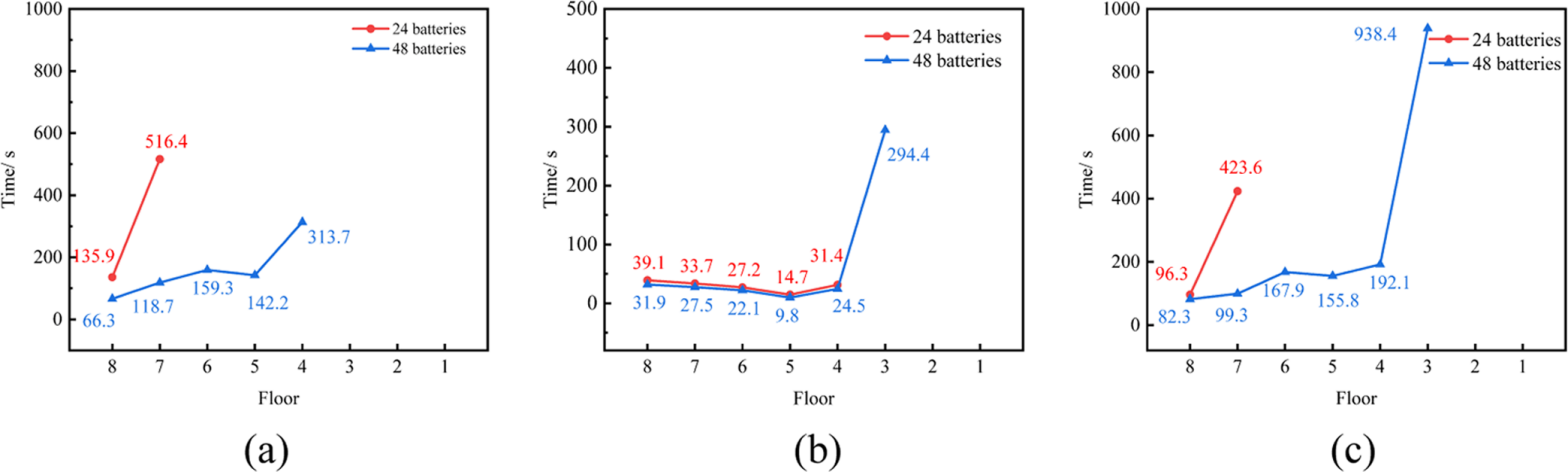
Time of TR gases released in different battery racks to reach explosion limit (where (a), (b), and (c) represent battery racks 1, 4, and 6).

Throughout the 1000 s diffusion process, the time taken for combustible gas at diverse locations to reach the explosion limit concentration varies. Figure 10 delineates the time taken for different positions within the 1, 4, and 6 battery clusters to reach the explosion limit when 24 and 48 batteries simultaneously undergo TR. The first two layers of the battery module within the battery cluster do not exceed the explosion lower limit, precluding ignition by open flames. In scenarios of simultaneous TR of 24 and 48 batteries, the fourth battery cluster is the earliest, where the combustible gas reaches the explosion limit, with the fifth layer being the foremost to reach the explosion limit at the quickest times of 9.8 and 14.7 s, respectively. Compared to 24 batteries in TR, 48 batteries in similar conditions reach the explosion limit more expeditiously across various time frames and different battery module layers within the battery cluster, with a greater number of battery module layers attaining the explosion limit.

**Figure 11:**
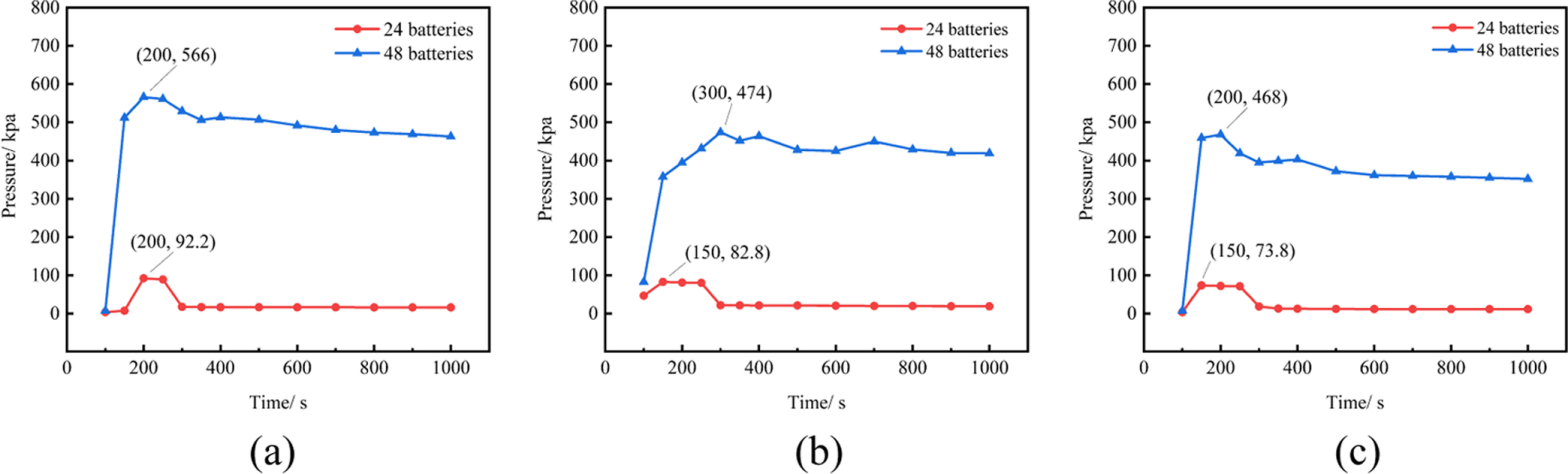
Changes of explosion overpressure in different ignition positions (where (a), (b), and (c) represent ignition positions 1, 2, and 3).

Explosion experiments are conducted within the diffusion–explosion model, utilizing gas diffusion data postbattery TR. The explosion overpressure exerted on the right-side door of the prefabricated cabin during the combustion expansion of flammable gas serves as the characterization parameter for the explosion simulation experiment. As observed from the gas cloud diffusion diagram in Figure 8, the concentration of combustible gas is highest in the upper space of the prefabricated cabin. Ignition is executed at positions 1–3 as depicted in Figure 5, and the resulting explosion overpressure at various times is illustrated in Figure 11. The peak explosion overpressure transpires between 150 and 310 s postgas diffusion initiation. The explosion overpressure at each location initially increases, subsequently decreases, and eventually stabilizes over time. When 24 batteries undergo simultaneous TR, the maximum explosion overpressure of 92.2 kPa is generated at the first ignition position at 200 s. The explosion overpressure induced by the combustible gas released by 48 batteries undergoing simultaneous TR markedly surpasses that of 24 batteries. The explosion overpressures generated at positions 1–3 in the middle of the prefabricated cabin with 48 batteries undergoing TR are 566, 474, and 468 kPa, respectively. The explosion at the first ignition position exerts the maximum pressure on the right-side cabin door, while the third ignition position exerts the minimum. Hence, the farther the ignition position is from the cabin door, the greater the force of the explosion on the cabin door.

### 3.3 Simulation of Battery TR at Various Locations

**Figure 12:**
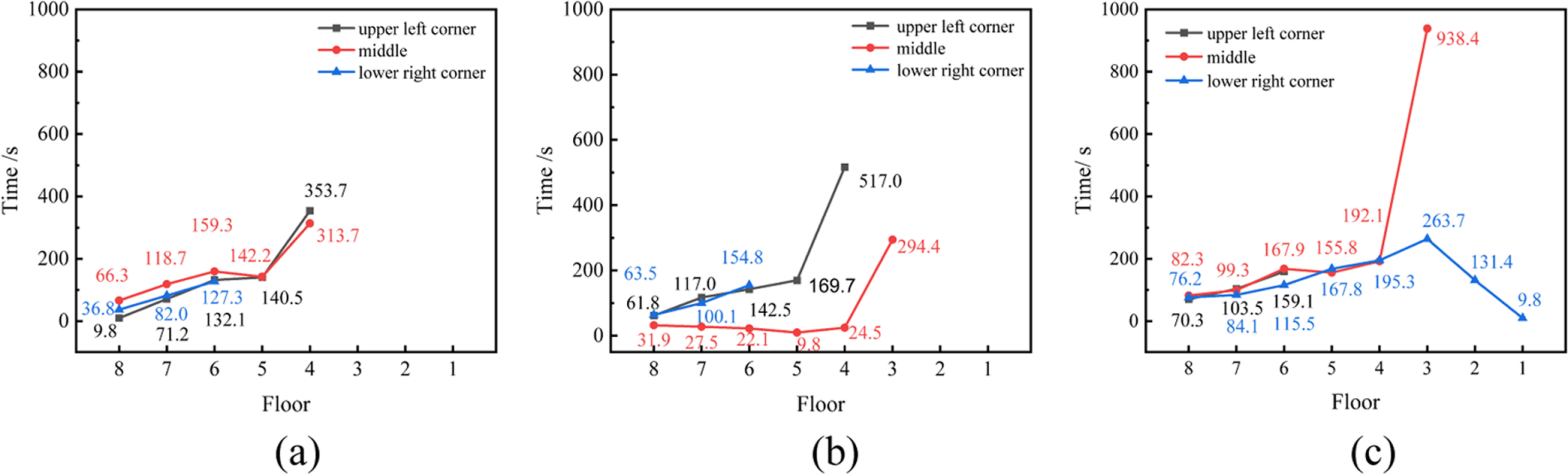
Time of TR gases released in different battery racks to reach explosion limit (where (a), (b), and (c) represent battery racks 1, 4, and 6).

Owing to the larger volume of gas cloud generated by the TR of 48 batteries and its more intricate and comprehensive distribution in the prefabricated cabin, the gas cloud data of 48 batteries undergoing simultaneous TR is selected, as depicted in Figure 4. Simulations are conducted at the upper left, middle, and lower right positions, respectively. The time distribution diagrams of the combustible gas concentration reaching the explosion limit in each layer of battery modules in the 1, 4, and 6 battery clusters during the diffusion process are shown in Figure 12. It is observed that the shortest time for the combustible gas concentration in the prefabricated cabin to reach the explosion limit is 9.8 s, regardless of the battery thermal runaway location.

**Figure 13:**
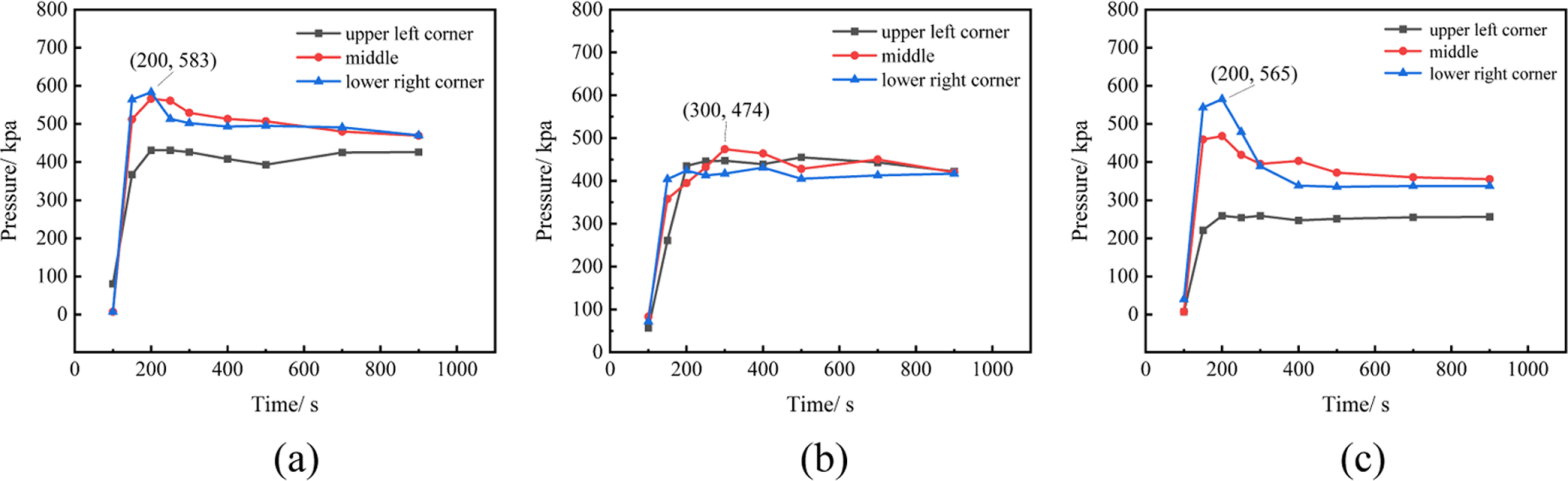
Change of explosion overpressure due to different ignition positions (where (a), (b), and (c) represent ignition positions 1, 2, and 3).

The aforementioned battery TR combustible gas cloud data are input into the diffusion–explosion model for simulation experiments. The changes in the explosion overpressure generated by the explosion are shown in Figure 13. The maximum overpressure generated by the explosion occurs at the first position, which is 583 kPa, indicating that the upper layer of the prefabricated cabin will incur more severe damage when the combustible gas is ignited. As observed from Figure 13, the maximum explosion overpressure generated when the ignition point is in the middle position of the prefabricated cabin is significantly smaller than that of the two side ignition points. Therefore, the middle position of the prefabricated cabin presents the least likelihood of serious harm during the 1000 s diffusion process of mixed combustible gas.

### 3.4 External Impact of Combustible Gas Explosion

**Figure 14:**
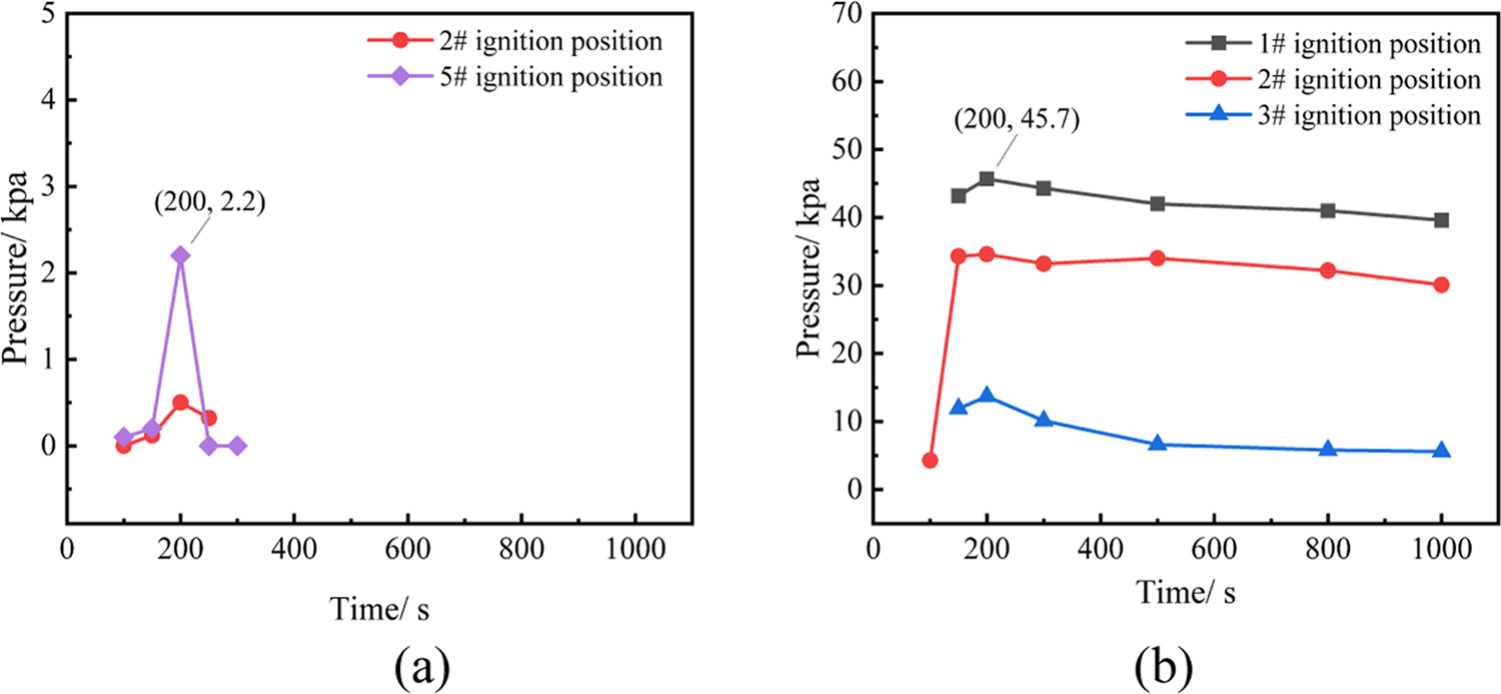
Explosive overpressure variation of 24 and 48 batteries TR at 5 m outside the hatch (where (a) and (b) represent 24 and 48 batteries, respectively).

A monitoring point is established 5 m outside the cabin door to study the external impact of the explosion of combustible gas generated by battery thermal runaway in the prefabricated cabin. The changes in the maximum explosion overpressure generated by the explosion of gas from 24 batteries undergoing TR in the middle position of the prefabricated cabin at a distance of five meters outside the cabin door are shown in Figure 14(a). It is observed that the farther the ignition position is from the cabin door, the greater the explosion overpressure generated by the explosion. The maximum explosion overpressure detected at a distance of 5 m outside the cabin door is 2.2 kPa, posing no threat to the human body.

The changes in the explosion overpressure generated by the explosion of combustible gas at various ignition positions when 48 batteries undergo TR at different positions in the prefabricated cabin at a distance of 5 m outside the cabin door are shown in Figure 14(b). The experimental results demonstrate that the maximum explosion overpressure detected at a distance of five meters outside the cabin door exceeds 40 kPa, which will inflict serious harm to the human body.

Because batteries still have the potential for TR, BESS still have the potential for hazardous conditions to occur. Because current BESS still have the potential for TR, there is still the potential for hazardous conditions to occur in BESS. According to the conclusion of this paper, if fans, pressure relief panels, and inert gas devices are added to the upper part of the storage compartment during the construction of BESS in the future, it will guarantee a safer and smoother operation of the BESS.

## 4 Conclusions

In this paper, the analysis of the gas component of the battery heat of the LFP was carried out, the simulation model was established in FLACS software, and the law of diffusion of the gas and the explosion of the gas in the storage battery cabin were studied by establishing the diffusion–explosion model.

(1) Without considering the electrolyte vapor and smoke, the explosion limit of the mixed combustible gas is 4.86–52.2%. When the TR occurs in the middle of the 48 batteries in the prefabricated chamber, the shortest time for the concentration of combustible gas to reach the explosion limit is 9.8 s.
(2) The maximum explosion overpressure caused by the explosion of combustible gas generated by the TR of 24 and 48 batteries in the middle of the prefabricated cabin is 92.2 and 566 kPa. Compared with the upper left corner and lower right corner, the TR of the battery in the middle of the prefabricated chamber is the least likely to cause serious harm.
(3) When the combustible gas produced by the TR of 24 batteries explodes at different locations in the prefabricated cabin, the maximum explosive overpressure generated at 5 m outside the prefabricated cabin door is 2.2 kPa, which does not pose a threat to the human body. However, when 48 batteries are thermally out of control, the maximum explosive overpressure can reach 45.7 kPa, which will cause serious harm to the human body.

## Supporting Information Available

Content of multiple battery thermal runaway tests was demonstrated and validation of the software simulation content was carried out (PDF)

## Notes

**Funding Sources** This research was funded by the special program for independent research of key technologies for fire prevention and control in electrochemical energy storage power stations (grant number: 2023YFC3009900).

## Nomenclature

SOC: state of charge
SOH: state of health
TR: thermal runaway
LFP: lithium iron phosphate
BESS: battery energy storage system
C: measurement of the charge current with respect to its nominal capacity
CH_4_: methane
CO: carbon monoxide
CO_2_: carbon dioxide
C_2_H_4_: ethylene
C_2_H_6_: ethane
C_3_H_6_: propylene
C_3_H_8_: propane
H_2_: hydrogen
ρ: time-averaged value of fluid density
φ: average value of a general variable
*u*: time-averaged value of speed
Γ: turbulent transport coefficient
*S*_φ_: source term of different φ items
*u*_*j*_: velocity vector of particles in the *j* direction
*x*_*j*_: j coordinate axis direction in the fluid
Γ_fu_: turbulent dissipation coefficient of fuel transmission characteristics
*m*_fu_: mass fraction of fuel gas
