# Supplementary material for: Simulation of Dispersion and Explosion Characteristics of LiFePO4 Lithium-Ion Battery Thermal Runaway Gases
Source: ACS Omega. 2024 Apr 4;9(15):17036–44. doi: 10.1021/acsomega.3c08709 (PMC11025091; doi:10.1021/acsomega.3c08709)
Supplement: Supplementary file 1 — ao3c08709_si_001.pdf [file ao3c08709_si_001.pdf]

SUPPLEMENTARY INFORMATION

The Simulation of Dispersion and Explosion Characteristics of $LiFePO_4$ Lithium-ion Battery Thermal Runaway Gases

Mingjie Zhang[1], Kai Yang[1], Qianjun Zhang[1], Hao Chen[1], Maosong Fan[1], Mengmeng Geng[1], Bin Wei[1] and Bin Xie[2]*

1 China Electric Power Research Institute, Beijing, CN 100192

2 Jescom Software (Shanghai) Co., Shanghai, CN 200090

*Corresponding Author: yangkai@epri.sgcc.com.cn

Tel.: +86-136-8132-3201

1. Battery thermal runaway gas production test

We performed thermal runaway (TR) tests on a 105Ah Li-$FePO_4$ battery with 100% SOC in the pressure vessel shown in Figure S1(a) in the manuscript. We chose two lithium iron phosphate batteries, numbered 1 and 2, and the experimental conditions were consistent with those described in the manuscript. Using the overheating method to trigger the TR of the battery, the battery TR experiment was carried out in a pressure vessel and the battery TR gases were collected and analyzed for gas composition using a gas chromatograph. During the TR of the battery, the pressure in the pressure vessel, the battery surface temperature and voltage changes are shown in Figure S1. The maximum temperature of the battery surface during TR is 401 °C and 378 °C, and the maximum pressure detected in the pressure vessel is 22 KPa and 23 KPa.

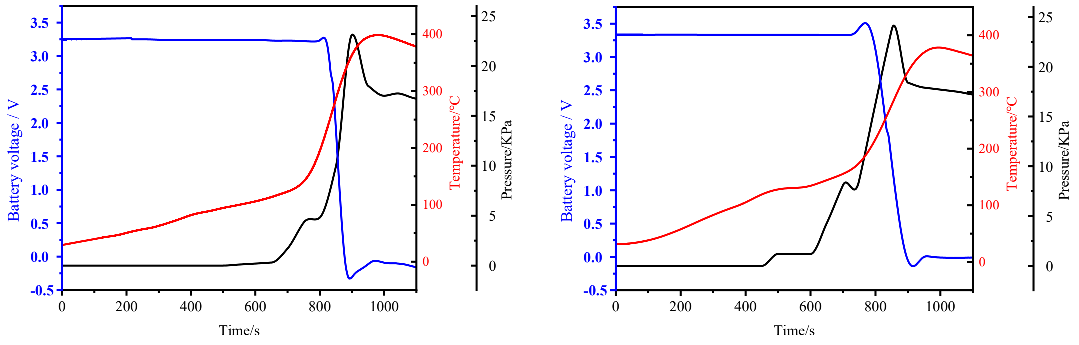

(a) 1# battery (b) 2# battery

Figure S1. Battery TR pressure, temperature and voltage variation graphs

The volume percentages of the main gases produced by thermal runaway of 105 Ah $LiFePO_4$ batteries measured using gas chromatography are shown in Table S1. By calculation, the proportions of various gases released from the TR of the battery in the three experiments were basically similar, and the error between gases of the same type did not exceed 10%. The volumes of gases released from the TR of the battery measured in the three experiments were 87.8 L, 81.6 L and 85.3 L, respectively.

The error in the volume of TR gas for batteries 1 # and 2 # compared to the volume of gas used in the experiment in the manuscript was 1.05 % and 4.34 %, respectively. Such experimental errors are within acceptable limits, so that the composition and volume data of the gases released from the TR of the battery in the manuscript can be simulated and tested in the FLACS simulation system.

Table S1. Battery thermal runaway gas volume fraction

Battery number	CH_4/%	C_2H_6/%	C_2H_4/%	C_3H_6/%	H_2/%	CO/%	CO_2/%	Other/%	Gas volume/L
1#	3.72	1.25	8.91	0.78	53.46	4.98	24.18	2.72	86.2
2#	3.99	1.38	9.35	0.55	50.09	4.43	22.55	7.66	81.6
manuscript	3.93	1.41	9.22	0.59	51.37	4.71	22.93	5.84	85.3

Due to the fact that BESS are complex internally and contain large amounts of energy, there may be a greater risk of safety-related experiments if they are done directly in the energy storage system, and they may also result in a greater loss of resources. Therefore, we are going to study the diffusion law and ignition law of the

mixed combustible gases released from the BESS when the TR of the battery occurs in the FLACS simulation software. Since FLACS simulation software has been more widely used in the petrochemical field, we believe that this software is able to help the researcher to accomplish some fundamental research. In order to verify the soundness of the simulation process we used, we also constructed a small model of an exploding sphere for simulation experiments and fabricated an identical physical entity in the laboratory.

We conducted simultaneous online simulations and offline experiments using a small exploding sphere model to study the explosion characteristics of a battery TR combustible gases, and examined and compared the explosion overpressure in the offline experiments and online simulations.

2. Explosive ball device experiment

In this experiment, the 20 L explosive sphere test device is used for explosion test of gas, as shown in Figure S2. The explosion sphere is made of double-layer 316 stainless steel, the inner diameter is 336 mm, consists of two parts: the sealing cap and the explosion chamber, which has good corrosion resistance and high temperature strength. Explosion sphere and vacuum pump, gas cylinder, ignition system is connected, the right side of the sphere is installed with a pressure sensor, for real-time monitoring of the pressure of the gas mixture explosion. Explosion sphere on both sides of the quartz glass visual observation window, pressure resistance of 4 MPa, to facilitate the high-speed camera to shoot images of the flame in the process of mixed gas explosion, before and after the window is parallel to each other, the two sides of the window axis is the same, through the center of the ball position.

The working temperature of the explosion sphere is room temperature, and the specification of the pressure sensor is 113B21SNLW42703, the range is 1 KPa ~ 3.09 MPa, and the charge sensitivity is 3.555 MPa / V. Before the experiment, the pressure sensor and the ignition device are loaded into the 20 L explosion sphere, and the explosion sphere is evacuated using a vacuum pump. Formal experiments, close the

evacuation valve, use the vacuum pump to evacuate the explosion ball, when the pressure in the explosion ball becomes - 0.08 MPa or less, fill with air, repeat the above steps three times. According to the partial pressure method, the use of vacuum pumps will explode the pressure in the ball down to the required partial pressure value, and then fill the gas mixture to the pressure gauge for 0 MPa, the pressure gauge range of - 100 KPa ~ 100 KPa. Ignition test to detect the gas explosion overpressure.

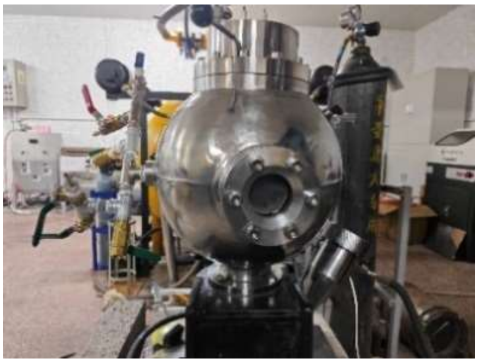

Figure S2. 20L explosion ball physical picture

105 Ah LFP TR gas and air mixture in the explosion dome for explosion experiments, the pressure sensor in the explosion dome wall monitoring the maximum explosion pressure, produced a fitting curve shown in Figure S3.

In the air for ignition experiments, the gas sensor to monitor the maximum pressure of 1.5 KPa; in the proportion of 5 % and 5.3 % of the mixture of gases, the maximum explosion pressure of 1.97 KPa and 2.53 KPa, in the gas explosion critical value. In the proportion of 6 % of the gas mixture, the maximum explosion pressure of 18.27 KPa, in the proportion of 14.00 % of the gas mixture, the maximum explosion pressure of 416 KPa, the maximum explosion pressure for the gas mixture explosion curve. The experimentally measured Lower Explosive Limit Concentration (LELC) of the gas mixture was 5.0 %, and the LELC of the gas mixture calculated by the Le Chatelier formula was 4.94 %, with an error of 1.2 %, which is within the expected error range.

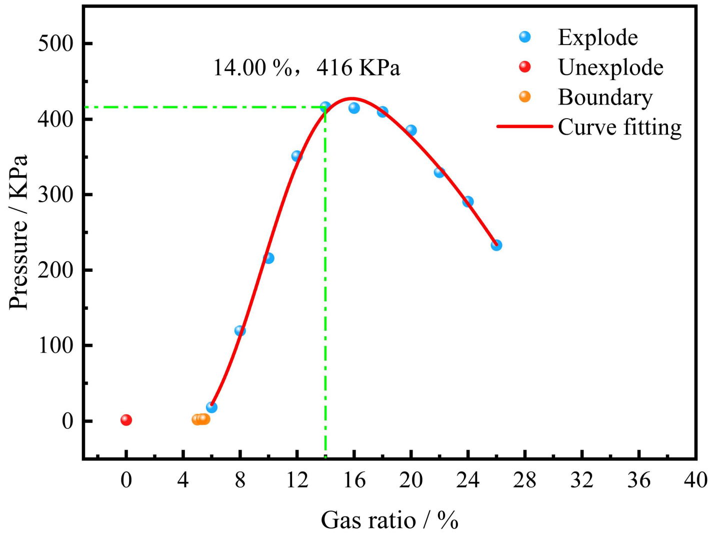

Figure S3. 105 Ah battery TR gas mixed with air explosion overpressure

3.FLACS Simulation Study

FLACS simulation experiments were conducted using the gas mixture composition used in the explosion ball experiment, the gas composition is the gas mixture generated by the TR of the 105 Ah LFP and air, and the gas composition is shown in Table S2.

Table S2. Composition of gas mixtures

Gas composition	Ratio/%
CH_4	3.93
C_2H_6	1.41
C_2H_4	9.22
C_3H_6	0.59
CO_2	22.93
CO	4.71
H_2	51.37
other	5.84
All	100

The gas released from the TR of 105Ah $LiFePO_4$ battery is filled into the explosion ball for simulation experiments, and the pressure change process in the explosion ball during the ignition process is monitored by the pressure sensor in the explosion ball. In the process of the experiment, two changes need to be observed, one is the process of the explosion ball in the combustible gas can be ignited, and the other is the explosion pressure generated after the ignition.

In FLACS simulation software, the 3D physical model of the 20 L explosive sphere is shown in Figure S4. The radius of the sphere in the model is 0.118 m, and the sphere is filled with a mixture of air and the gas released from the TR of 105 Ah LFP, which is filled with different proportions of gases for the ignition test. The various parameters used in the explosion ball simulation model and the BESS simulation model are identical.

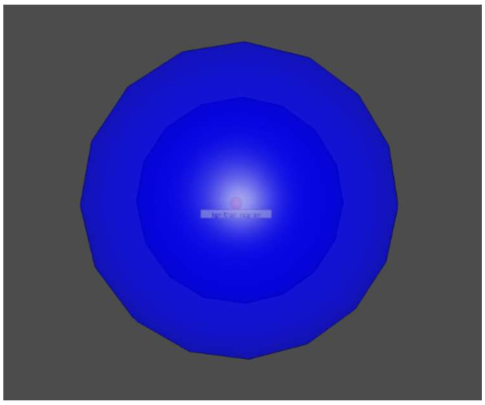

Figure S4. 3D model diagram of 20 L explosive ball

The explosion simulation curve of the gas mixture of the battery TR release and air in the explosion ball is shown in Figure S5, and the curve is the fitting curve of the explosion simulation results. The results show that, with the proportion of combustible gases released from the battery TR in the gas mixture increases, the explosion pressure increases and then decreases, and the explosion pressure reaches a maximum of 450 KPa when the proportion of mixed gases is 16.00%.

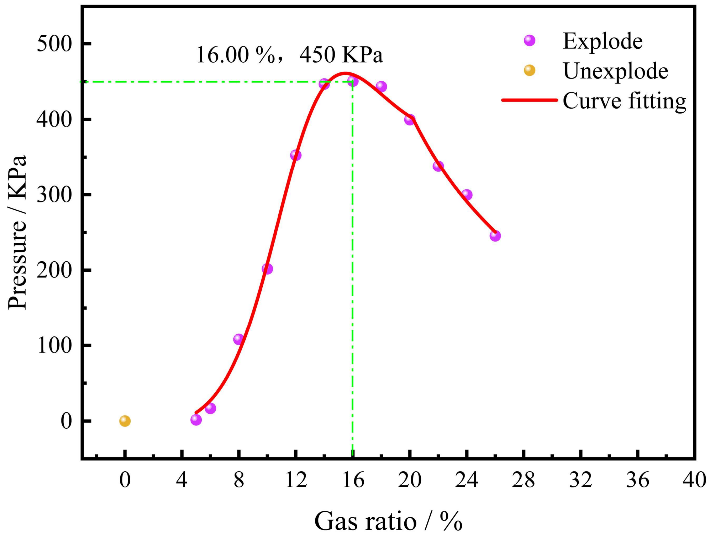

Figure S5. Simulation curve of battery TR gas explosion in the explosion ball

4.Differences between offline experiments and simulation results

Battery thermal runaway gas released in the explosion ball explosion simulation curve and experimental comparison shown in Figure S6, the curve for the explosion pressure of the fitted curve, the results show that the gas explosion experiment measured in the explosion pressure curve of the maximum explosion pressure of 416 KPa, the gas explosion simulation of the explosion pressure curve measured in the explosion pressure of the maximum explosion pressure of 450 KPa. The error between the 20L exploding ball experimental results and the exploding ball simulation results is 8.17%, this result is within the error range.

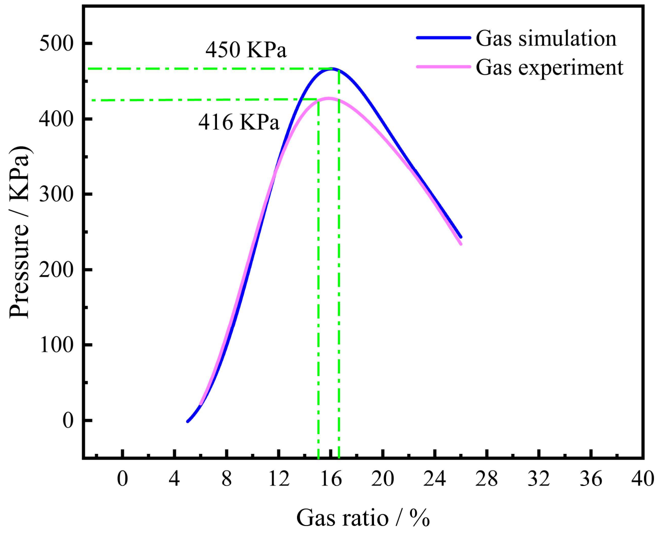

Figure S6. Comparison between simulation and experiment of battery TR gas in the explosion ball

5.Conclusion

According to the comparison between the offline 20 L explosion ball experiment and the explosion ball simulation experiment, we find that the FLACS simulation software is able to simulate basically similar experimental results to the offline experiment in the small model constructed, and the error between them is within a reasonable range. Therefore, we can conclude that the simulation model of the battery storage system constructed in FLACS software is capable of simulating the simulation experiment of the gas mixture ignition released by the thermal runaway of the battery.
